# A systemic review on development of mesoporous nanoparticles as a vehicle for transdermal drug delivery

**DOI:** 10.7150/ntno.77395

**Published:** 2023-01-01

**Authors:** Praveen Kolimi, Sagar Narala, Ahmed Adel Ali Youssef, Dinesh Nyavanandi, Narendar Dudhipala

**Affiliations:** 1 Department of Pharmaceutics and Drug Delivery, School of Pharmacy, University of Mississippi, Oxford, MS - 38677, USA.; 2 Department of Pharmaceutical Technology, Faculty of Pharmacy, Kafrelsheikh University, Kafrelsheikh 33516, Egypt.

**Keywords:** Mesoporous silica nanoparticles, surface functionalization, tunable pore size, biocompatibility, biodistribution, safety, toxicity

## Abstract

Recent advances in drug delivery technologies utilizing a variety of carriers have resulted in a paradigm shift in the current approach to diagnosis and therapy. Mesoporous silica nanoparticles (MSNs) were developed in response to the need for materials with high thermal, chemical, and mechanical properties. The synthesis, ease of surface functionalization, tunable pore size, large surface area, and biocompatibility of MSNs make them useful in a variety of biomedical applications such as drug delivery, theranostics, and stem cell research. In addition, MSNs have a high capability of delivering actives ranging from small molecules such as drugs and amino acids to larger peptides, vaccines, and antibodies in general. Moreover, MSN-based transdermal delivery has sparked a lot of interest because of the increase in drug stability, permeation, and ease of functionalization. The functionalization of MSNs plays an important role in the efficient delivery of therapeutic agents in a highly controlled manner. This review introduced dermal and transdermal drug delivery systems, explained the anatomy of the skin, and summarized different barriers that affect the transdermal delivery of many therapeutic agents. In addition, the fundamentals of MSNs together with their physicochemical properties, synthesis approaches, raw materials used in their fabrication, and factors affecting their physicochemical properties will be covered. Moreover, the applications of MSNs in dermal and transdermal delivery, the biocompatibility of MSNs in terms of toxicity and safety, and biodistribution will be explained with the help of a detailed literature review. The review is covering the current and future perspectives of MSNs in the pharmaceutical field with therapeutic applications.

## Introduction

### Introduction about dermal and transdermal delivery

There has recently been a surge of interest in novel drug delivery systems for existing drug molecules. The novel drug delivery system improves patient compliance while increasing the safety and efficiency of a drug molecule 1. Dermal (topical) drug delivery is used to define localized action to the pathological sites within the skin with minimal systemic absorption. However, transdermal drug delivery systems (TDDS) are defined as self-contained discrete dosage forms that deliver active(s) through the skin at a controlled rate into the systemic circulation over an extended period of time.

The first Food and Drug Administration (FDA, 1979) approved TDDS was a three-day patch for scopolamine (Transderm-Scop) to treat motion sickness 2. A decade later, nicotine patches were the first transdermal blockbuster, which increased awareness of transdermal delivery among medical professionals and the general public. Nowadays, there are more than 19 TDDS for many actives including, estradiol (Estraderm), nitroglycerin (Transderm-Nitro), fentanyl (Duragesic), clonidine (Catapres-TTS), lidocaine (Lidoderm), and testosterone (Testoderm). Moreover, fixed dose combination patches containing more than one active for contraception and hormone replacement e.g., estradiol with norethidrone (Combipatch), and iontophoretic (fentanyl HCl/Ionsys) and ultrasonic (lidocaine/SonoPrep) delivery systems for analgesia were developed 2.

TDDS is one of the primary research areas for third-generation pharmaceutical preparations, alongside oral medication, and injection. The reasons for this are found in the drug's administration route, which is convenient, easy to use, non-invasive, improves patient compliance, and is appropriate for patients who are unconscious or vomiting, as well as those who rely on self-administration 3,4. Because TDDS does not involve gastrointestinal passage, there is no drug loss due to first-pass metabolism, and drugs can be delivered without interference from pH, enzymes, or intestinal bacteria. Another benefit of TDDS is that the dose is reduced when compared to the oral dosage forms for the same drug 5. Moreover, TDDS improved bioavailability, more uniform plasma levels, and longer duration of action, resulting in lower dosing frequency, fewer side effects, and improved therapy due to plasma level maintenance until the end of the dosing interval, as opposed to a decline in plasma levels with conventional oral dosage forms 5-7.

The protective function of human skin places physical and chemical constraints on the type of permeant that can pass through this barrier. Drugs must have sufficient lipophilicity and a molecular weight of less than 500 Da to be delivered passively through the skin. Because of these requirements, the number of actives, available for the manufacture of transdermal or dermal products is limited 8.

The following characteristics should be present in an ideal drug candidate for transdermal drug delivery 1:

- The molecular weight should be <500Da;
- Log partition coefficient (log P) should lie within a range of 1-3;
- The drug molecule should be potent with a therapeutic dose of less than 10 mg;
- The aqueous solubility should be greater than 100 ug/mL.

There are two types of TDDS 2,6:

- First-generation; transdermal patches (e.g., Transderm^®^ Scop and Catapres-TTS 1) are medicated adhesive patch that is applied to the skin to deliver a specific dose of medication through the skin to the bloodstream.
- Second-generation formulations expand the scope of transdermal drug delivery to enhance the permeability of the barrier. However, permeation enhancement methods such as conventional chemical enhancers, iontophoresis, and non-cavitational ultrasound, have battled for the balance between achieving improved delivery across the stratum corneum and protecting deeper skin tissues from damage 5.

#### The anatomy and physiology of the skin

With a surface area of 1.7 m^2^, the skin is the largest organ of the body, accounting for nearly 16 percent of an average person's total body mass 9,10. The primary function of the skin is to protect the body from microorganisms, Ultra-violet radiation permeation, chemicals, allergens, and water loss 11.

**Figure 1:**
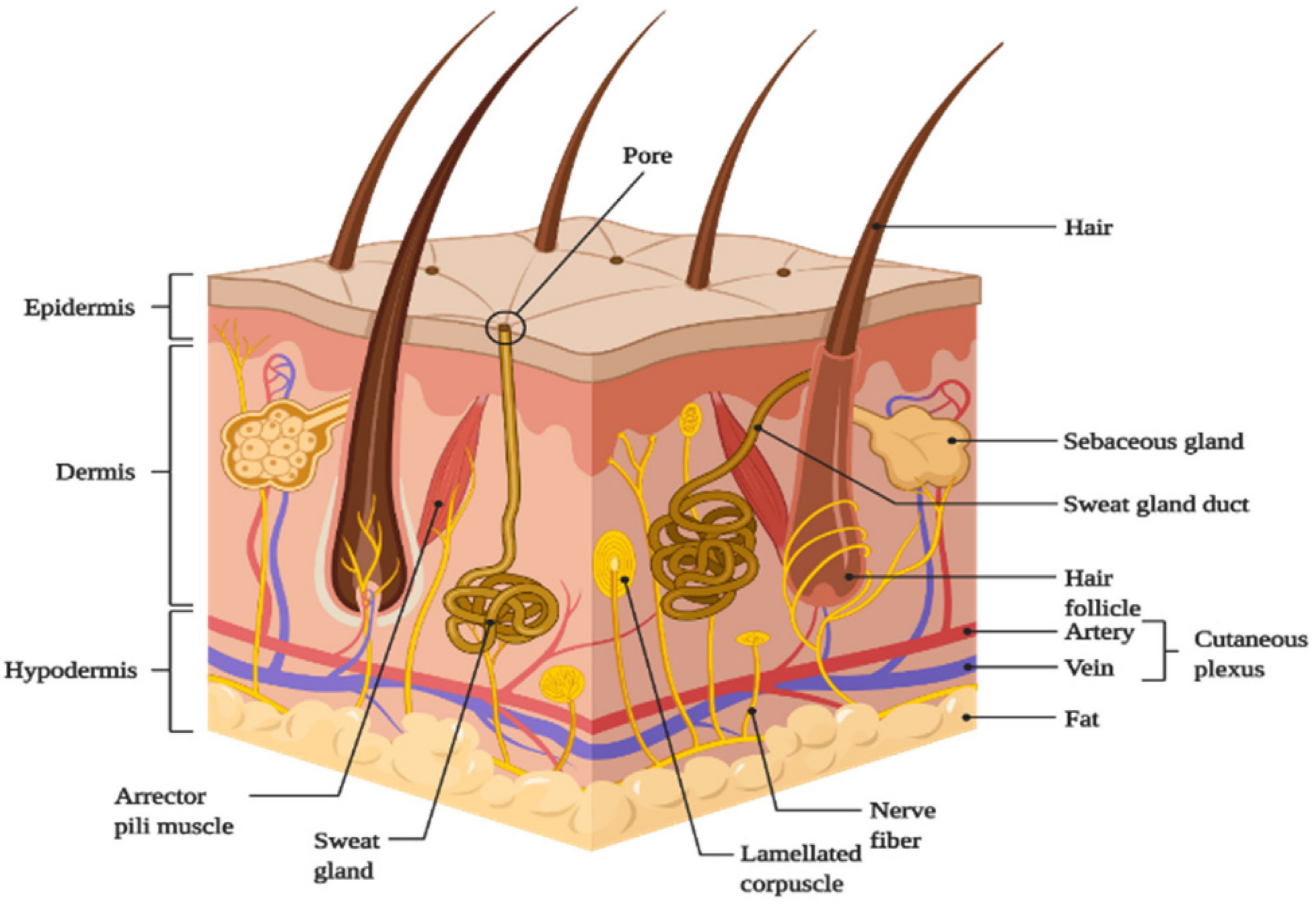
Schematic representation of basic human skin anatomy depicting different skin layers and their components (created with Biorender.com).

The skin is divided into three main regions (Fig. 1):

- Outermost layer- Epidermis;
- Middle layer- Dermis;
- Innermost layer- Hypodermis or Subcutaneous tissues.

##### Epidermis

This is the skin's outermost layer, also known as the horny layer, and has a thickness of about 0.8 mm 1. From the inside to the outside, the epidermis has five layers: the stratum basale, stratum spinosum, stratum granulosum, stratum lucidum, and stratum corneum (SC) 5. The epidermis is organized in the form of 'bricks' and 'mortar', with protein-rich keratinocytes acting as 'bricks' and intercellular lipids acting as 'mortar' 4. The stratum corneum, which is composed of metabolically active agents such as mitochondria and ribosomes, serves as the primary barrier to drug penetration 1,3-5,11.

##### Dermis

It has a thickness of 3-5 mm. The dermis is made up of collagenous (70%) and elastic fibers, which give the skin strength and elasticity. Both the dermis and the epidermis receive nutrition from blood vessels 1,3. It also has nerves, macrophages, and lymphatic vessels. The dermis is metabolically active and important in regulating body temperature, wound repair, delivering oxygen and nutrients to tissue, and removing waste products 4,5,11.

##### Hypodermis

The hypodermis or subcutaneous fat tissue layer supports the dermis and epidermis 10. It is the layer of skin that connects to the underlying tissues of the body, such as muscles and bone. As a result, the main functions of the hypodermis are physical shock protection, heat insulation, and support and conductance of the skin's vascular and neural signals 5. It serves as a reservoir for high-energy molecules and transports the major blood vessels and nerves to the skin. Fat cells in the subcutaneous layer account for approximately 50% of total body fat 3.

#### Drug penetration routes

**Figure 2:**
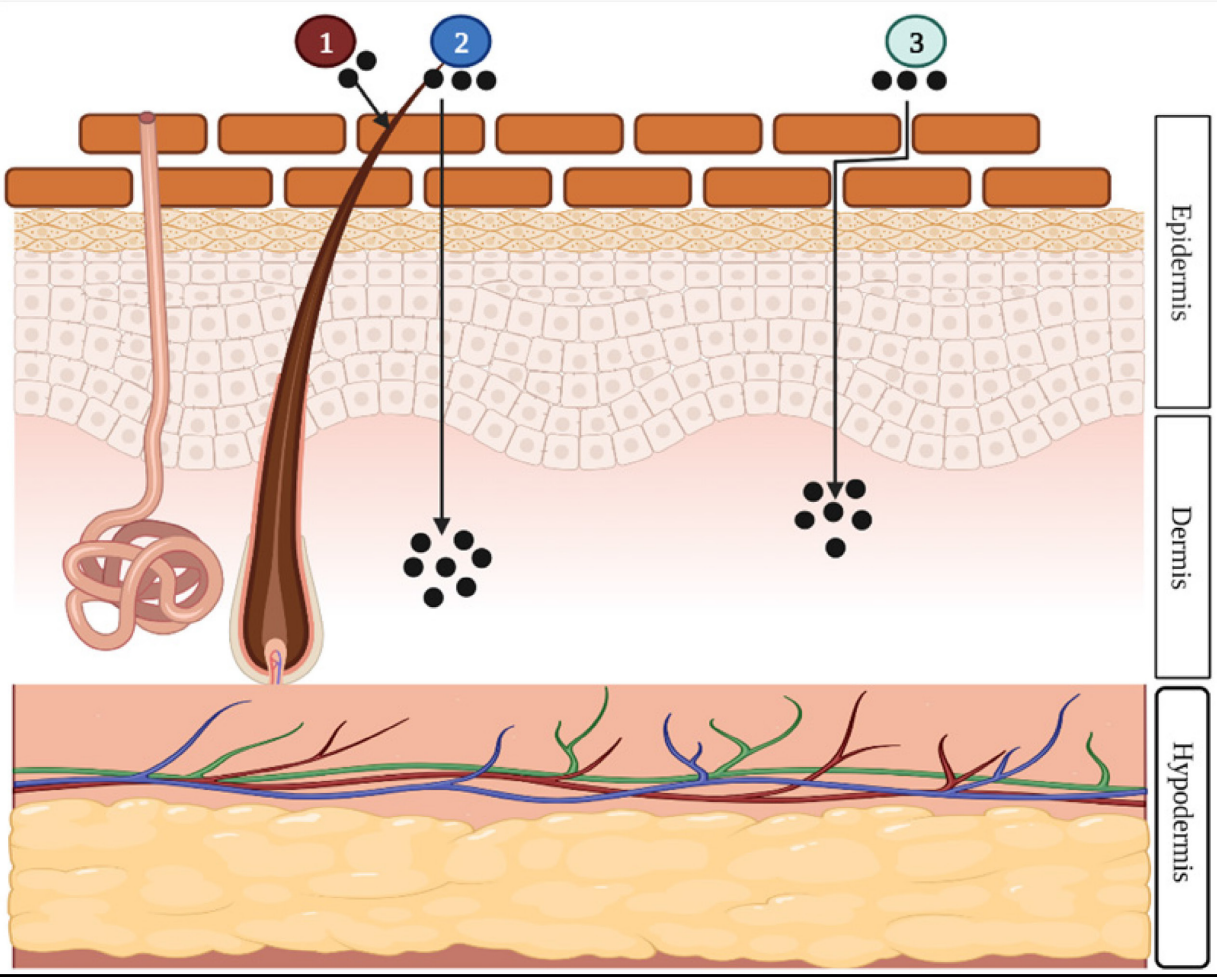
Illustration of the three different drug penetration pathways: (1) the transappendageal route, (2) the intracellular route, or (3) the intercellular route. The appendageal route is drug penetration through hair follicles, sweat glands, or skin furrows. The intracellular is drug penetration through the cell membrane of the epidermal cells. The intercellular is drug penetration between epidermal cells 13 (created with Biorender.com).

There are mainly two routes of dermal and transdermal delivery of drugs: transepidermal (intercellular and intracellular) and transappendageal (follicular) pathways (Fig. 2). The transepidermal route involves the passage of the molecules through the stratum corneum whereas the passage of molecules through sweat glands and across the hair follicles takes place in the case of the transappendageal route 3,4,12.

### Various barriers to the transdermal drug delivery

There are various biological and physiological factors affecting the skin barrier and hence transdermal delivery of the drug.

Biological factors 13-19:

- **Skin condition:** Injury to the stratum corneum that disrupts its continuity increases permeability due to increased vasodilation caused by the removal of the barrier.

- **Lipid Film:** The lipid film on the skin's surface acts as a protective layer, preventing moisture loss from the skin and assisting in the maintenance of the stratum corneum's barrier function. Transdermal absorption was found to be reduced when this film was defatted.
- **Skin Hydration:** Although the degree of penetration enhancement varies from drug to drug, hydrating the stratum corneum can improve transdermal permeability. Skin hydration can be achieved by simply covering or occluding the skin with plastic sheeting, allowing sweat and condensed water vapor to accumulate. Increased hydration appears to open the skin's dense, tightly packed cells and increase porosity.
- **Skin age:** young skin is permeable compared to elder skin. Toxins are more easily absorbed through children's skin. Thus, one of the factors influencing drug penetration in TDDS is skin age.
- **Blood supply:** Transdermal absorption can be influenced by changes in the peripheral circulation.
- **Regional skin site:** The thickness of the skin, the nature of the stratum corneum, and the density of appendages differ from one location to the next. These elements have a significant impact on penetration.
- **Skin metabolism:** Steroids, hormones, chemical carcinogens, and some drugs are metabolized by the skin. As a result, the efficacy of drugs permeated through the skin is determined by skin metabolism. Catabolic enzymes found in the viable epidermis may render a drug inactive through metabolism, affecting the drug's topical bioavailability.
- **Skin temperature:** The human body maintains a skin temperature of 32-37 °C. As a result, increasing the temperature causes an increase in diffusion through the tissue.
- **pH:** only unionized (neutral) molecules pass easily across the lipid membrane, and weak acids and bases ionize to different degrees based on their pH and pKa or pKb values. Consequently, the concentration of unionized species will determine the effective membrane gradient, which is pH-dependent.
- **Species differences:** The thickness of the skin, the density of appendages, and the keratinization of the skin vary between species, influencing penetration.

#### Physiochemical factors

##### Physiochemical properties of active moiety 11,14-19

- **Partition coefficient:** The drug is soluble in both water and lipids. Log K 1-3 is the ideal partition coefficient for intermediate transdermal delivery. The intracellular route is preferable for highly lipophilic drugs (log k 43), whereas the transcellular route is preferable for hydrophilic drugs (log k 51).
- **Molecular size:** small molecules penetrate faster than large molecules, so molecular weight has an inverse relationship with drug absorption.
- **Diffusion coefficient:** The diffusion coefficient of the drug affects drug penetration. The diffusion coefficient of a drug at a constant temperature is determined by the properties of the drug, the diffusion medium, and their interaction.
- **Ionization:** According to the pH-Partition hypothesis, unionized drug permeates the skin.
- **Solubility/melting point:** At normal temperature and pressure, most organic solutes have a high melting point and low solubility. Lipophilic drugs permeate faster than hydrophilic substances, but they must also be water-soluble to some extent, as is required in most topical formulations.

### Role of nanoparticles in transdermal drug delivery

Nanoparticulate carriers are colloidal particulate systems with a size range of less than 500 nm 20. Nanoparticulate carriers can be used to modify the physicochemical properties of drugs as well as their interactions with physiological systems. The use of nanoparticulate carriers for skin delivery is particularly important because it not only overcomes the limitations of traditional delivery systems but also improves drug permeation through the skin 21. Nanoparticulate carriers can improve drug transport across the skin by ensuring direct contact with the stratum corneum and skin appendages, controlling drug release, increasing contact time with the skin, and protecting drugs from physical and chemical instabilities. The use of nanoparticles (NPs) is one of the passive strategies to enhance the delivery of drugs through transdermal delivery. Liposomes, transfersomes, ethosomes, niosomes, dendrimers, lipid, and polymer NPs, and nanoemulsions are the most used and researched nanocarriers for dermal/transdermal drug delivery in the pharmaceutical industry. The advantages and disadvantages of using nanocarriers for transdermal drug delivery are, in general, their small size, high surface energy, composition, architecture, and attached molecules 12,22.

#### Liposomes

Liposomes are microscopic lipid-based vesicles made of cholesterol, non-toxic surfactants, sphingolipids, glycolipids, long-chain fatty acids, and even membrane proteins into which nutrients or drugs can be loaded for delivery 23. Liposomes, solid lipid NPs (SLNs), and nanostructured lipid carriers (NLCs) are all examples of lipid-based formulations currently available. Because of their biocompatibility and biodegradability, lipid-based systems are less toxic than other drug delivery systems (DDSs) like polymer NPs 24. Liposome-like vesicles, such as niosomes, transfersomes, and ethosomes, have been proposed to overcome some of the limitations of liposomes by varying in lipid composition and preparation method.

#### Niosomes

Niosomes are non-ionic surfactant-based vesicles composed primarily of non-ionic surfactants and cholesterol 26. They have good chemical stability during storage and can overcome many of the drawbacks associated with liposome delivery, such as high costs and variable phospholipid purity. Niosomes increase the drug's residence time in the stratum corneum and epidermis as well as its permeation into the deeper layers of the skin 12,27.

Maheshwari et al. investigated the use of niosomes as carriers for topical delivery of vaccines using hepatitis B surface protein as an antigen and the non-toxic cell-binding B subunit (CTB) of cholera toxin B as an adjuvant. *In vitro* permeation and skin deposition studies revealed that hepatitis B surface protein-loaded niosomes formulation had deeper skin permeation than conventional liposomes and plain antigen solution. Furthermore, topically applied hepatitis B surface protein-loaded niosomes to Balb/c mice elicited a strongly systemic and mucosal humoral immune response, demonstrating the antigen encapsulated niosomes/adjuvant formulation's potential as a novel vaccination strategy 28.

#### Transfersomes

Transfersomes are thought to be the first generation of highly elastic or deformable vesicles. They are a new type of liquid state vesicle composed of phospholipids and an edge activator, which is typically a single chain surfactant (e.g., sodium cholate, span 60/65/80, and tween (20/60/80) 29,30. Cevc et al. studies the effect of Transfenac, a topical diclofenac transfersomes formulation, and outperformed a commercial hydrogel in mice, rat, and pig models to treat moderate pain, signs, and symptoms of osteoarthritis or rheumatoid arthritis. When compared to a commercial hydrogel, diclofenac associated with ultra-deformable transfersomes has a longer duration of action and concentrations in tissues under the skin that are ten times higher. Furthermore, the system was able to penetrate deep into soft tissue, and a sustained release from carriers deposited in subcutaneous tissue was observed 31.

#### Ethosomes

Ethosomes are lipid vesicular carriers that contain ethanol in relatively high concentrations for improved drug permeation through the skin. They are primarily made up of phospholipids, ethanol, and water. The high ethanol concentration, which distinguishes ethosomes from other vesicular carriers, promotes skin permeation and the release of entrapped drug particles into deeper layers and systemic circulation 22.

Dayan et al. developed and studied trihexyphenidyl HCl ethosomal formulations (THP). It was discovered that ethosomes have a high encapsulation efficiency and an excellent ability to deliver the drug to the deeper layers of the skin 32.

#### Dendrimers

Dendrimers are highly branched polymers with a highly symmetric spherical shape that are chemically produced and have a diameter of 1-10 nm 34. They're usually made from sugars, nucleotides, and amino acids, which are either natural or synthetic. Hydrogen bonds, electrostatic interactions, and hydrophobic interactions could trap drugs in the dendrimer core 22. Hegde et al. investigated the use of chemically conjugated drug-peptide dendrimers in the presence of iontophoresis as a topical formulation to improve the transdermal permeation of ketoprofen 35. Passive diffusion, sonophoresis, and iontophoresis-assisted penetration of four peptide dendrimer-drug conjugates (D1-D4) across mouse skin were studied. The *in vitro*/*in vivo* studies revealed that sonophoresis and/or peptide dendrimers are not suitable approaches to enhance the permeation of ketoprofen, plain ketoprofen has been delivered to a greater extent than the conjugates, and a therapeutical concentration of ketoprofen can be transdermally delivered only with the application of electric current to D2 conjugate 35.

#### Lipid NPs

##### Nanostructured lipid carriers (NLC)

NLCs are colloidal carriers with a solid lipid core composed of a mixture of solid and liquid lipids and a mean particle size in the nanometer range 37. They are made up of a lipid matrix with a unique nanostructure. By providing an imperfect crystal, increased drug solubility in a mixture of solid and liquid lipids significantly improves drug encapsulation efficiency in nanostructured lipid carriers and reduces drug expulsion 38. Shah et al. investigated the effect of polyarginine chain length on the topical delivery of surface modified NLCs. The surface modification of nanostructured lipid carriers with a peptide containing 11 arginine moieties significantly improved the transport of spantide II (SP) and ketoprofen (KP) to the deeper skin layers, resulting in a reduction of inflammation associated with allergic contact dermatitis in mice model 39.

##### Solid-lipid nanoparticles

Solid lipid nanoparticles (SLNs) are made up of a lipid matrix, which is a biodegradable raw material that is physiologically tolerated 40,41. They are good candidates for transdermal delivery because they can be prepared in various sizes and the surface polarity can be modified to improve skin penetration 12,22. Liu et al. prepared and characterized SLNs of diclofenac sodium by modified emulsion/solvent evaporation method. It was reported that the SLN formulation of diclofenac sodium showed improved dermal localization with an entrapment efficiency of 89% and drug loading of 9.5% 42.

##### Nanoemulsion

Nanoemulsions are isotropic dispersed systems of two non-miscible liquids, typically consisting of an oily system dispersed in an aqueous system, or an aqueous system dispersed in an oily system but forming nanometric-sized droplets or other oily phases 29. They are non-toxic and non-irritant systems that can be used for skin or mucous membranes, as well as parenteral and non-parenteral administration in general 22,43. Many drugs have been dermally and transdermally delivered by various nanoparticulate systems (Table 1) 12,22.

##### Mesoporous silica nanoparticles (MSNs)

Because of their inherent properties, such as stability, tunable porosity, mesoporous nature, biocompatibility, ease of functionalization, non-toxicity, and biocompatibility, MSNs have been developed to serve as theranostic probes and versatile drug delivery systems (DDS) 45. MSNs have a honeycomb-like chemical structure (porous) and a large active surface area, allowing different functional groups to be attached to target the drug moiety to a specific site 46,47.

## Origin of MSNs

**Figure 6:**
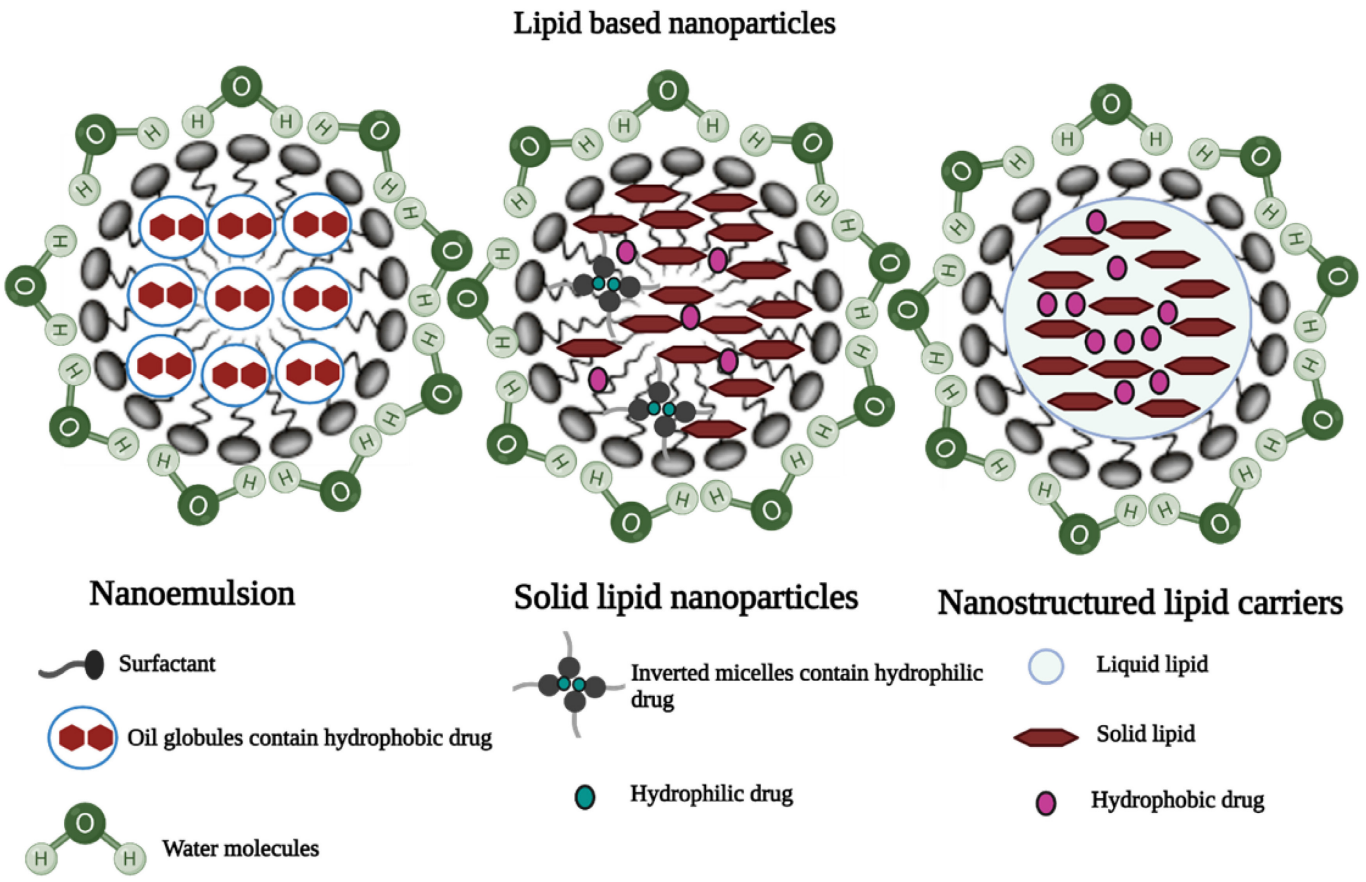
Illustrations of three different lipid-based nanoparticles: **a)** Nanoemulsion, **b)** Solid lipid nanoparticles, and **c)** Nanostructured lipid carriers 44 (created with Biorender.com).

Although mesoscopic materials have been synthesized since the 1970s, Mobil Research and Development Corporation was the first to use a liquid crystal template mechanism to create mesoporous solids from aluminosilicate gels in 1992. It was given the designation MCM-41 (Mobil Crystalline Materials or Mobil Composition of Matter) 45,48. The mesoporous form of silica has special properties, especially when it comes to loading large amounts of therapeutic agents and releasing them. Silica-based mesoporous NPs are more stable to external responses such as degradation and mechanical stress due to a strong Si-O bond. The mesoporous structure can be tailored to the size and type of drugs by changing the pore size and porosity of the structure. The pore geometrics of mesoporous structures are shown in Fig. 6. Table 2 shows some different types of MSNs including, the internal structure and pore size.

## Physio-chemical properties of MSNs

The MSNs have numerous features such as controlled particle size, porosity, morphology, high chemical stability, well-established drug delivery research, and versatility for creating high-performing hybrid materials 55,56.

### Size

While MSN size is primarily controlled before administration, it is important to keep in mind that physiological reactions *in vivo* can cause significant changes in nano-vehicle size 57.

### Shape and porosity

Physiological fate and *in vivo* MSNs behaviour are both influenced by the final shape of the MSNs core. The most common shapes for MSNs are spheres and rods. The final shape is primarily determined by the cosolvent's identity and volume ratio relative to water during the sol-gel reaction 57,58. When compared to solid and mesoporous silica counterparts, MSNs with rough surfaces showed increased uptake by target cells 57,58.

The shape, diameter, and number of pores in MSNs define porosity. The cosolvents used during synthesis play a great role in determining the shape of the pores. When strong bases like NaOH are used as the cosolvent, the standard honeycomb pore shape is produced. When other cosolvents, such as triethanolamine (TEA), are added to the synthesis reaction, wormhole pores form 59. The honeycomb MSNs has less restricted pore space and more stable colloidal suspension as compared to wormhole MSNs 57. Also, honeycomb MSN releases drugs in a less controlled manner 57.

### Surface properties, charge, and toxicity

To deliver the encapsulated drug to a specific site of action, the surface of MSNs can be modified by adding a stimuli-sensitive gatekeeper. The addition of gatekeeper molecules to the surface of MSNs provides cargo specific release while alleviating toxicity concerns. Silanol, which has the general form Si-OH, is the primary functional group found on unmodified MSNs 60. At physiological pH of 7.4, hydrogen-containing silanol groups can deprotonate, leaving MSNs surface with a net negative charge 61. Negatively charged NPs are less likely to interact with or be engulfed by nonphagocytic cells, allowing them to circulate for longer time period 62,63. However, the benefit of the negative charge comes at the cost of an increased risk of hemolytic interactions between MSNs and red blood cells. Moreover, the charges also affect immune cells and function negatively 57,64,65. MSNs can be coated with biocompatible materials to render them non-toxic for safe use for various biomedical applications 66.

## Synthesis of MSNs

### Synthesis approaches

The synthesis process of MSNs starts with the replication of a surfactant liquid crystal structure and a subsequent polymerization of metal oxide precursors 46. Then, the removal of the surfactant by the calcination process forms a porous structure 46.

#### Solution approach

Mobile crystalline material (MCM-41) is the most widely used MSNs. MCM-41 consists of hexagonal arrangement of cylindrical mesopores 46. The templating of an alkyl ammonium salt, Cetyl trimethyl ammonium bromide, is necessary for the synthesis of this type of MSNs 67,68. The first step is adding high concentration of surfactant to an aqueous solution polysilicic acid or silica acid (precursor) to form micelles. The second step is the electrostatic and interaction and hydrogen bonding between the precursor and the hydrophilic interface to form an amorphous silica, which is the mold of the mesoporous product. The third step is removing the remaining surfactant by calcination and extraction method 46.

#### Sol-gel Process

**Figure 7:**
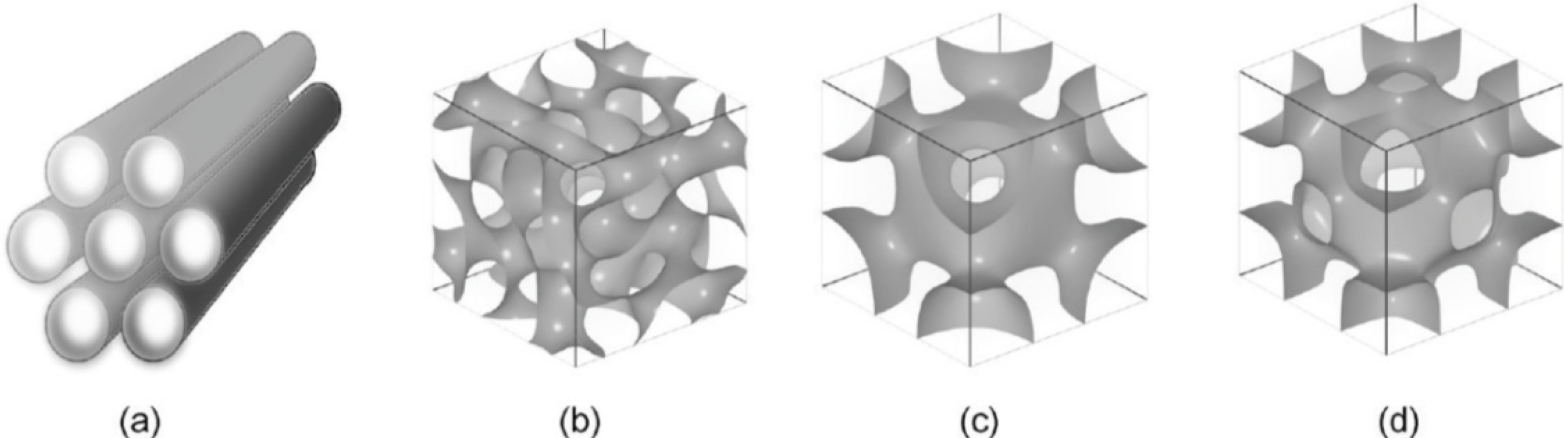
Illustrations of the different structures found in mesoporous materials: (**a**) hexagonal, (**b**) cubic (Ia3d), (**c**) cubic (Im3m), and (**d**) cubic (Pm3m) 54.

To fabricate MSNs with controlled mesopore structure and surface properties, a simple process known as the “sol-gel process” is usually used. This procedure does not require many excipients and is not a multi-step process. Therefore, a low-cost synthesis procedure can be adopted to prepare MSNs 47. Hydrolysis and condensation reactions are the two major steps in the Sol-gel process. Hydrolysis can be stimulated in acidic or alkaline pH to produce colloidal particles in an aqueous solution 46. Then, the cross-linking of sol particles through siloxane bonds results in a gel-like three-dimensional (3D) network at neutral pH that can speed up the condensation reaction. The condensation step is reversible and the silica can be easily restructured 45,47,48. Depending on the structure and porosity of MSNs, different biomolecules can be embedded in the matrix of silica gel for controlled release applications after drying at ambient temperature as shown in Fig. 7 47. The MCM-41 MSNs can be prepared in the size range of 60-1000 nm by this process. This process has several advantages, including being a simple and cost-effective method for providing MSNs with controlled mesoporous structure and surface properties 45.

#### Evaporation-induced self-assembly (EISA)

This method was introduced in 1997. Throughout the process, all the reactants undergo concentration changes due to evaporation. The silica precursor is organized into a liquid-crystal-like template as a result of this 48. The soluble silica and surfactant (critical micelle concentration) were dissolved in a mixture of ethanol and water. Then, solvent evaporation process starts during dip coating to increase surfactant concentration 62. By injecting it into an aerosol generator, it was converted to monodisperse droplets. The orifice of the generator can be changed to control the size of the final product. The formation of micelles and the co-assembly of silica and surfactant into liquid-crystal mesophases are induced by the evaporation of alcohol during drying 70. Evaporation-induced self-assembly is a non-volatile component that can be added to an aerosol droplet containing MSNs 45. The main parameters required to be considered during MSNs production are silica precursor, additives as well as the effect of temperature 47.

### Organically modified silica precursors

They do not undergo hydrolysis due to the presence of an organic group attached directly to the core silicon atom, which does not require oxygen bridge 46. Organo-silica NPs have superior properties, such as a larger surface area, a less condensed siloxane structure, and a lower density 46,71. Organic templates are only used in a few practical applications due to their limited accessibility and high cost 46. Glycerol-derived polyol-based silanes, orthosilicic acid, sodium metasilicate, tetraethyl orthosilicate (TEOS), or tetramethoxysilane (TMOS), and tetrakis (2-hydroxyethyl) orthosilicate are the most commonly used silica precursors 45,49.

#### Glycerol-derived polyol-based silane precursors

They are not pH sensitive, but they are very sensitive to the ionic strength of the solution. This use of this precursor results in monolithic MSNs that are optically clear. Because residuals can be removed or retained, shrinkage during long-term storage can be reduced 46. Because of the extensive time consumption and requirement of freshly prepared acid, the use of Orthosilicic acid as a silica precursor is not recommended anymore 72.

#### Sodium metasilicate

Sodium metasilicate can be used as a precursor to sol-gel-derived silica. However, salt removal (sodium chloride) from the product by dialysis process is a time consuming and costly procedure 73. Therefore, alkoxides and pure alkoxysilanes are widely used currently 46.

### Catalyst

To produce MSNs with the desired characteristics, the additives must carefully be selected. A surfactant and catalyst are the two main chemical components during MSNs production. When synthesis takes place at room temperature, the surfactant is usually required. When the temperature is below 25 °C and the pH is around 6, fibrous aggregates can form, which necessitate the use of surfactants. Increasing the temperature or changing the pH can disintegrate these aggregates, but it can also affect the loaded therapeutic agent(s) 74. Furthermore, selecting the appropriate surfactant is critical because it can improve the function of drug loading and release by facilitating a complex interaction between drug molecules and the matrix 75,76. Moreover, different pore size can be obtained depending on the surfactant chain length 75,76. The catalyst is another crucial component in the development of mesopore channels. Another important factor in the formation of mesoporous channels is the catalyst. Because hydrolysis and condensation are pH-dependent, different reactions can be catalyzed or inhibited by adding hydrochloride or sodium dioxide 47.

### Temperature

The high temperature has adverse effects on the properties of the prepared MSNs. The high temperature of spray drying after the sol-gel process results in non-porous MSNs 77. At high temperatures, mesopores shrink significantly, making it impossible to control the morphology of MSN and preventing the template from being recovered or re-used, resulting in high cost. Moreover, this failure can release noxious gases that can cause environmental issues 78,79.

### Functionalization of MSNs

MSNs have a well-defined structure and a dense layer of surface silanol groups that can be modified with a variety of organic functional groups 47. MSNs can be surface functionalized in different ways such as co-condensation and post-synthesis grafting 80. The surface functional groups can play several roles in biomedical applications of MSNs including, controlling the surface charge of MSNs, linking chemically with functional molecules inside or outside the pores, and controlling the size of the pore entrance for entrapping molecules in the nanopores. Moreover, the functionalization with organic groups could control drug absorption and release 47,56.

#### Post-synthesis grafting

**Figure 8:**
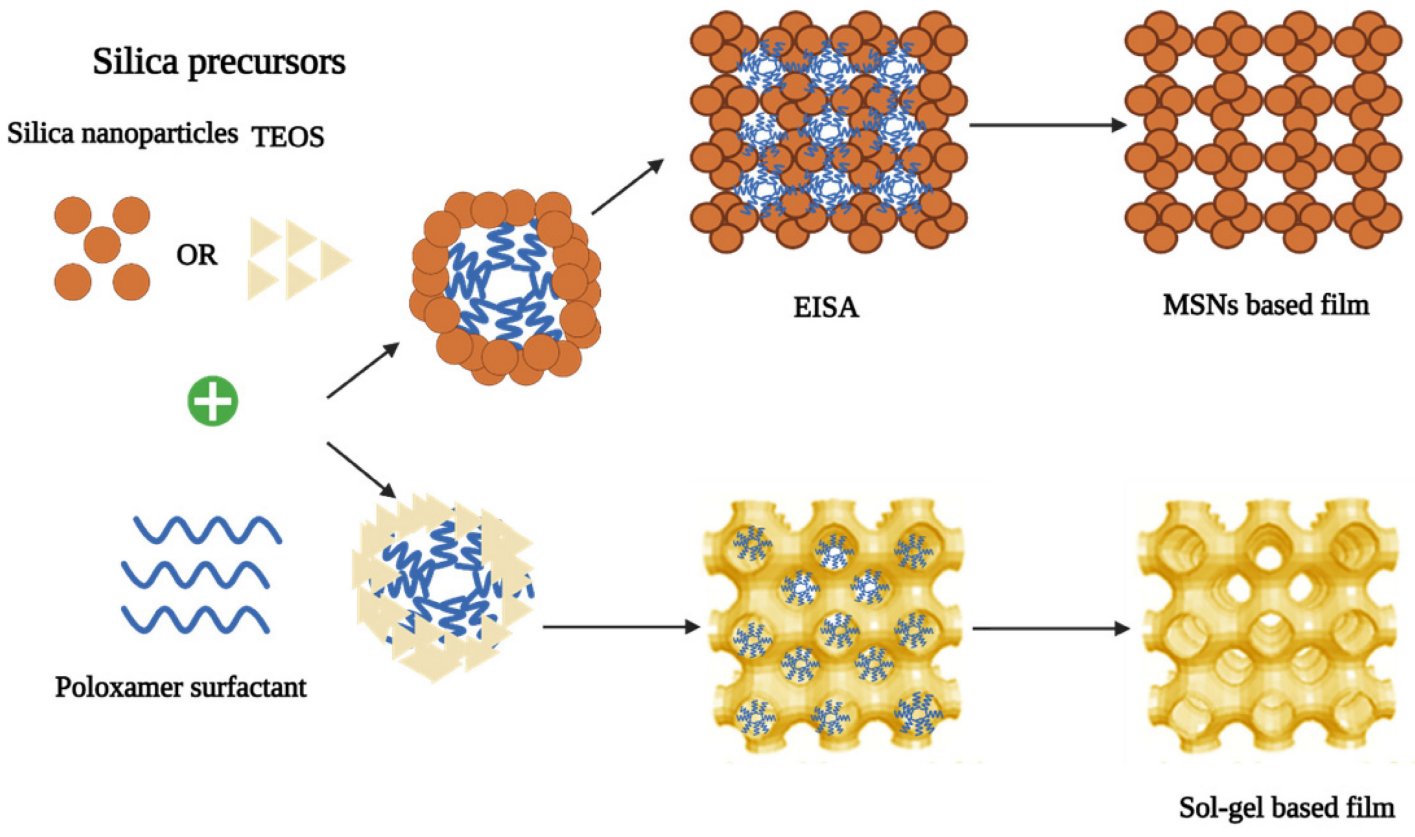
Synthesis of sol-gel and nanoparticle-based mesoporous silica films via Evaporation Induced Self-Assembly (EISA), using surfactants as template and TEOS or preformed silica nanoparticles as inorganic precursors 69 (created with Biorender.com).

This is the most common method for functionalizing MSNs, which involves attaching functional groups to the surface of a prefabricated inorganic mesoporous material, usually after the surfactant has been removed. Surface silanol groups (Si-OH), which can be present in high concentrations, act as convenient anchoring points for organic functionalization when grafting mesoporous silicates. Silylation is the most common method for surface functionalization with organic groups via grafting. Fig. 8 depicts the MSNs functionalization 80,81.

#### Co-condensation

The co-condensation method is another approach to make organically functionalized mesoporous silica materials. The co-condensation functionalization method is a direct synthesis method in which the organoalkoxysilane is added to a basic aqueous solution containing Cetyltrimethylammonium bromide (CTAB) and TEOS during condensation 55. The surfactant molecules can then be removed via ion exchange with an ethanolic ammonium nitrate solution 80. By adding functional co-condensing reagents to this synthetic approach, it is possible to control the morphology of the NPs 56.

## Factors affecting the characteristics of MSNs

### Control of particle size

Additives such as alcohols, amines, inorganic bases, and inorganic salts can be used to effectively control particle size. The hydrolysis and condensation of silica precursors can be altered by these agents. These additives can speed up the reaction kinetics, which results in smaller particles 48. The pH of the reaction medium is also crucial in determining the size of the fabricated MSNs. Ikari et al. discovered that the grain size of MSNs obviously depended on the cetyltrimethylammonium chloride (CTAC; cationic surfactant) and NH_4_OH concentrations. The MSNs size grew up to several hundreds of nanometres with relatively high NH_4_OH and low CTAC concentrations. However, an increase in CTAC and a decrease in NH4OH concentrations reduced the particle size (10 nm) 83. The presence of excess positively charged CTA^+^ was a result of a decrease in the concentration of NH_4_OH, which reduced the pH and thus the rate of hydrolysis of TEOS, and thus the concentration of silicate anion. Excess CTA^+^ coats the composite particles, reducing particle size in the same way that CTA^+^Cl^-^ does. The deposition of CTAC as neural species (CTA^+^Cl^-^) on CTA-silicate composite, which restricts particle growth, was thought to be the cause of the smaller particle size as the concentration of CTAC increased 83,84. The results were consistent with Yamada et al. who discovered also that increasing the cationic surfactant to silica ratio reduced the particle size of MSNs. This was explained by the fact that the nucleation rate of mesostructured material outnumbers the rate of particle growth. The formation of smaller particles is favoured by a high nucleation rate 83. In addition, Chiang et al. found that the particle size increases with the amount of TEOS. When different tetraalkoxysilanes with different alkoxy groups (Si(OR)_4_, R = Me, Et, Pr, and Bu) were used, the particle size increased. Alcohols also affected the particle size of the MSNs. This could be due to a change in the rate of hydrolysis 85.

### Control of Pore Size, Pore Volume, and Mesostructural Ordering

The chain length of surfactant plays an important role in determining the pore size and pore volume of MSNs. MSNs with larger pores is synthesized with longer chain length surfactant whereas short-chain length surfactant produces MSNs of small pores. Expander compounds like 1, 3, 5-trimethylbenzene, or linear hydrocarbons can also be used to increase the size of the pores 48,86. Whereas decreasing the concentration of surfactant increases pore wall thickness 86. The mesostructure ordering of the particles was influenced by the concentration of TEOS. A higher concentration of TEOS resulted in a disordered mesostructured, while a lower concentration was insufficient to form a mesoporous structure 85. The concentration of the surfactant CTAB had a significant impact on the particles' mesostructure arrangement 87. A lower surfactant concentration fails to form micelles, resulting in template-deficient NPs, whereas a high CTAB concentration may result in a disordered structure 87.

### Control of shape

The morphology of MSNs was found to be affected by the molar concentrations of surfactant, water, base catalyst, and TEOS. Cai et al. used TEOS, NaOH/NH4OH, and CTAB to create MSNs with a variety of shapes, including spherical, silica rods, and micrometre-sized oblate silica 48. Han et al. synthesized a wide range of MSN particles by controlling the amount of dodecanol used as a soft template and the synthesis temperature. The size, porosity, interior spaces, and shell structure of the six different particles were all controlled. The inclusion of dodecanol as a soft template resulted in particles with various morphologies, according to their findings 88.

## Advantages and challenges of the MSNs as nanotherapeutics

### Advantages

Some of the advantages of MSN are listed below 66,89-93:

- MSNs could be used as controlled drug delivery systems to keep drug concentrations at optimal levels for long periods, improving therapeutic outcomes while avoiding potential toxicity and side effects.
- MSNs can protect therapeutic agents during their journey through the body when they are used for drug delivery. Any potential cargo degradation would be avoided in this way, which is particularly important when delivering soft therapeutic agents like RNAs or proteins.
- Because MSNs have such a large loading capacity, they can transport two or more drugs into the same nanoparticle, allowing for the development of combined therapies for multi-resistant tumors.
- MSNs allows precise control of drug release thus avoiding a premature release of therapeutic agents which could result in toxicity.
- The functionalization of MSN makes it an ideal candidate for designing multifunctional nanosystems, boasting eligible qualities for high drug loading and gradual release.
- Due to its characteristics like tunable size, high surface area, well-ordered internal mesopores, large pore volume, high drug loading, good biocompatibility, and low production cost, MSNs can be used in many biomedical applications.

### Disadvantages 89,94,95

- The commercialization of MSNs under Good Manufacturing Practices (GMP) necessary for preclinical screening, clinical trial, and use is a major roadblock in the use of MSNs as nanotherapeutics.
- The synthesis of MSNs is relatively easy to repeat on a small scale, but it is much more difficult to control batch to batch on a larger and industrial scale.
- The potential toxicity and immunogenicity of MSNs which greatly depend on their surface functionalization is also a point to be considered from the biological point of view. Hence more human trials need to be conducted to test the toxicity and clinical efficacy of the agent.
- If the MSNs under investigation are to be used for cancer treatment, biodistribution should be thoroughly examined, as it is necessary to demonstrate that MSNs reach tumor tissue preferentially.
- Different parameters such as size-MSNs with 30 to 100 nm diameters can induce inflammatory responses in animal models-morphology, porosity, and surface charge-anionic surfaces are generally less toxic than cationic surfaces, which can cause hemolysis-, and functionalization affect MSNs degradability and toxicity. As a result, fine-tuning of these structural characteristics is required to develop safe silica nanocarriers.
- A lack of specific guidelines or requirements from regulation agencies for planning clinical trials for nanomedicines is also one of the challenges to translating nanomedicines from the lab to the clinic level.

## Application of MSNs in dermal and transdermal delivery

According to Nigro et al., the application of MSNs as a skin drug delivery system can be divided into three categories: dermo-cosmetic, biomedical, and cancer treatments 96-99. Transdermal drug delivery, gene delivery, and transcutaneous vaccination are some of the other applications 100,101. MSNs can be considered a viable strategy in the cosmetics industry to avoid the toxicity issues associated with sunscreen inorganic compounds (ZnO and TiO_2_). Furthermore, the incorporation of natural active agents into MSN pores can help to improve the stability of labelled natural active agents 96.

Ugazio et al. developed thermoresponsive MSNs as a nanocarrier for skin delivery of the well-known antioxidant quercetin 102. Two different types of MSNs, with pore size of 3.5 nm (MSN small) and 5.0 nm (MSN big) were prepared and physiochemically characterized. The biocompatibility was tested on a human keratinocyte cell line (HaCaT). The release profiles and *ex vivo* accumulation and permeation through porcine skin were also investigated. When comparing MSN small to MSN big, the quercetin loading was found to be higher in MSN small which could be due to the large pore size couldn't hold drug molecules efficiently. In comparison to bare MSNs, functionalized MSNs had a slower quercetin release, indicating a stronger drug-matrix interaction. In addition, an *in vitro* quercetin release assay was performed at temperatures below (20 °C) and above (40 °C) lower critical solution temperature (LCST), demonstrating that polymer chains respond to temperature stimuli by releasing flavonoid molecules immobilized within the mesopores 102.

Lio et al. developed a topical formulation of MSNs for the transdermal delivery of small interfering RNA in the treatment of facile skin cancer treatment 103. The MSN-oligonucleotide complexes were built on MSN of pore size 4 nm and thereafter coated with a layer of poly-L-lysine (PLL) to improve transdermal delivery of siRNA. The MSNs-PLL were tested with molecular beacon as the model oligonucleotide on human squamous cell carcinoma *in vitro*. MSNs-PLL enabled the delivery of TGF-β R siRNA during the regulation of gene expression in the tumor xenograft model. Based on these findings, MSNs-PLL could be used as a viable topical platform for non-invasive transdermal drug delivery 103.

Rizzi et al., conjugated MSNs with the second-generation photosensitizer verteporfin, and the resulting nanoplatform (Ver-MSNs) were tested in an *in vitro* photodinamic therapy model as a potential tool for melanoma treatment 104. Based on the findings of Endocytic Uptake Inhibition Studies in the presence of endocytosis inhibitors, an attenuation of Ver-MSNs based photodinamic therapy induced cell death, and a recovery in cellular morphology was observed 104.

Nafisi et al. prepared lidocaine (local anesthetic) inclusion complexes with MSNs (MCM41) and studied the effect of surface functionalization with positively charged amino-propyl groups to improve lidocaine permeation into the skin. The complexes were prepared in different lidocaine/MSNs ratios (3/1, 2/1, 1/1). The lead lidocaine-loaded MSNs (1/1) formulation showed higher drug release rates and skin permeation over pure lidocaine and bare lidocaine/MCM41 which can be due to the electrostatic interaction between positively charged functionalized lidocaine/MCM41 and the negatively charged skin cells. The current study demonstrated the potential of Lido/MN41-NH2 in the biomedical field, particularly for dermal drug delivery 105.

Ionic liquid are organic salts present in a liquid state at room temperature, while deep eutectic solvents are mixtures of hydrogen donors and acceptors with a melting point below room temperature. Both species share low volatility, low flammability, thermal and electrochemical stability, and good solvation ability 106. Ionic liquids and deep eutectic solvents do not rely on geometrically confined transport pathways and were effective in improving the transdermal delivery of macromolecules 106. Zhao et al., developed a non-invasive strategy for the transdermal delivery of MSNs using deep eutectic solvent from amino acid and citric acid. MSNs were surface modified by citric acid and then reacted with Lysine to form the deep eutectic solvents-MSNs. The intradermal and transdermal penetration assays were used to show if the deep eutectic solvents-MSNs could synchronously drive the MSNs to penetrate across the entire skin via a “Drag” effect or not. The transdermal delivery of the MSNs into blood circulation through topical application was successfully achieved. This work was promising and would extend the application of the MSNs and provide a novel strategy for the extended delivery of MSNs for better therapeutic outcomes.

Xu et al., designed a novel microneedles-based delivery device integrated with insulin loaded and H_2_O_2_-responsive MSNs to achieve fast and painless administration 107. The MSNs was prepared by modification by 4-(imidazoyl carbamate)phenylboronic acid pinacol ester and following a host-guest complexation between this compound and α-cyclodextrin. Insulin and glucose oxidase (glucose-responsive factor) were loaded into the MSNs. Glucose oxidase in MSNs could convert glucose to gluconic acid and generate H_2_O_2_. Then, the phenylboronic ester on the surface of MSNs could be oxidize in the presence of H_2_O_2_ that resulted in the destruction of host-guest complexation, leading to disassemble of the drug loaded MSNs and subsequent release of the preloaded insulin. After the transdermal administration of MSNs to the diabetic rats, an effective hypoglycemic effect was achieved. This work suggests that novel microneedles-based delivery device integrated with insulin loaded and H_2_O_2_-responsive MSNs could have a promising application in diabetes.

## Biocompatibility and biodistribution of MSNs

### Toxicity and safety

Nanoparticles' safety and toxicity are a major source of concern due to their high surface-to-volume ratio compared to their counterparts. The biocompatibility is a requirement for any pharmaceutical product to ensure that it does not accumulate in the body over time and cause unwanted effects 48. Several types of MSNs are nontoxic in a variety of biological systems when prepared with certain optimized structural features and administered at the appropriate dosages 108. Although these promising results in terms of their safety for human use have been obtained, there are some drawbacks, such as stability issues, and the difficulty in overcoming certain biological barriers associated with these carriers. Despite non-toxic properties, MSNs are still the most widely investigated nanocarriers 45,48.

### Effect of Surface Chemistry, Shape, and Size of MSNs

MSNs' fate is determined by their size, shape, pore order, and surface chemistry. MSN size is frequently cited as one of the most important factors in determining whether MSNs have desired or unintended effects on biological systems, i.e., whether MSNs are biocompatible or highly toxic when they come into physical contact with biological entities 48. Furthermore, it has been demonstrated that the size and other structural features of nanomaterials influence how they interact with cells or some cellular processes. The relationship between the sizes of a given nanocarrier, such as MSNs, and their biological activity, on the other hand, cannot be fully discussed without mentioning the nanoparticles' dosage 108.

Vallhov et al. studied the effect of MSN size on cellular uptake and function 109. Two anionic surfactant-templated mesoporous silicas (AMS) were synthesized by using calcination to remove the two templates. The biological effects of MSNs were investigated by incubating NPs with human dendritic cells (DC). When MSNs with similar surface areas were compared, those with smaller sizes or lower dosages had a smaller impact on DC immune functions, cell viabilities, and particle uptakes than those with larger sizes or higher dosages. Although the MSNs with various sizes entered the DCs via similar mechanisms, the larger ones were found to escape from the endolysosome with greater ease than the smaller ones 109.

In another study, Qianjun et al. prepared and evaluated the biodistribution and urinary excretion of MSNs of different sizes in Institute of Cancer Research (ICR) mice to investigate the effect of particle size and PEGylation. They reported that MSNs and PEGylated MSNs of various particle sizes (80-360 nm) are mostly found in the liver and spleen, with a small percentage in the lungs and a few in the kidney and heart. PEGylated MSNs with smaller particle sizes escaped the capture by the liver, spleen, and lung tissues more easily, had a longer blood-circulation lifetime, and showed slow biodegradation, resulting in a lower amount of degradation products excreted in the urine. Moreover, neither MSNs nor PEGylated MSNs resulted in any *in vivo* tissue toxicity after one month 110.

Zhao et al. recently discovered that the larger SBA-15 type MSNs (531 nm) were engulfed by RBCs more readily and caused greater membrane distortion in the cells than their smaller MCM-41 type (122 nm) counterparts 111. The larger SBA-15-type MSNs induced a stronger local membrane deformation, which resulted in more particle internalization and, ultimately, more hemolysis. The smaller MCM-41-type MSNs, on the other hand, were adsorbed onto the surface of RBCs without disrupting their membrane or morphology 111.

Malfanti et al., developed MSNs as vehicles for the delivery of the antitumoral drug gemcitabine (GEM) and its lipophilic prodrugs-4-(N)-acyl derivatives, (4-(N)-valeroyl-(C5GEM), 4-(N)-lauroyl-(C12GEM) and 4-(N)-stearoyl-gemcitabine (C18GEM)-for physical and a chemical protection of GEM from rapid plasmatic metabolization 112. The MSNs with/without grafting with aminopropyl and carboxyethyl groups were prepared and characterized. GEM was not loaded in any MSNs, while C12GEM was efficiently encapsulated and used for further evaluation. The surface functionalization of MSNs improved the loading capacity and hydrophobic and hydrogen bonding interactions with functional groups (and related alkyl chains) are played an important role in the interaction with the lipophilic prodrugs hosted in the MSN pores. The cytotoxicity studies in different cancer cell lines- MDA-MB-231 (human breast adenocarcinoma) and A2780 (human ovarian carcinoma) cells at different time (24, 48, and 72 h)-showed that C12GEM loaded MSNs were less cytotoxic than the free drug with an activity that increased with the incubating time, indicating that MSNs were able to release the drug in an extended manner. The results revealed that these MSNs could be an interesting system for the delivery of anticancer drugs.

#### Effect of surface chemistry

The main toxicity pathway associated with silica is due to its surface chemistry (silanol groups), which can interact with membrane components, causing cell lysis and cellular component leakage 113,114. In comparison to non-porous silica, mesoporous silica had a lower hemolytic effect. The biodistribution and biocompatibility of MSNs are also influenced by their surface properties. The MSNs can avoid being captured by liver, spleen, and lung tissues through changing their surface features by functionalization with PEG 48. Slowing et al., studied the effect of surface chemistry of MSNs on their cellular uptake and subsequent endosomal escape by synthesizing different MSNs by functionalizing MCM-41 with 3-aminopropyl (AP), guanidinopropyl (GP), 3- [N-(2- guanidinoethyl) guanidine] propyl (GEGP), and N-folate-3-aminopropyl (FAP) group 115. All functionalized MSNs had lower surface area and pore volume compared to parent MCM-41. It was observed that the FAP-MCM-41 MSNs were engulfed by both clathrin and foliate receptor-mediated endocytosis, followed by more confinement of positively charged particles inside endosomes. The parent MCM-41 MSNs were ingested through a clathrin-mediated pathway, whereas the AP- and GP-grafted MCM-41 MSNs were internalized into cells via a caveolae-dependent mechanism. The effects of the surface properties MSNs, such as surface electrochemistry, on cellular uptake and subsequent endosomal escape, were demonstrated in these findings 115.

Townson et al. performed an experiment in which PEG-PEI and PEG-NMe_3_^+^ MSNs were created by modifying MSNs of the same size, porosity, and charge with appropriate reagents to ensure exposed polyamines and distributed, obstructed amine groups, respectively. To determine the toxicity effects, both *in vitro* and *in vivo* experiments were carried out. The cytotoxicity of the synthesized NPs was tested on a variety of cell lines which showed that PEG-PEI MSNs were found to bind to all the cells whereas PEG-NMe_3_^+^ MSNs showed limited binding 116.

Paris et al., successfully designed and prepared polymer-grafted MSNs as ultrasound-responsive drug nanocarriers for the anticancer drug doxorubicin 117. The authors developed a nanocarrier with nanogates that allows the encapsulation and transportation of doxorubicin with no premature release to specific locations in the body where the drug can be released upon externally applied ultrasounds. MSNs induced no toxicity at least up to 500 μg/mL in LNCaP cells as measured by MTS reduction assay after incubation with tumor cells (LNCaP cells, from human prostate adenocarcinoma) for two hours. The MSNs were endocyted by LNCaP cells retaining their ultrasound-responsible capability because the NPs only induced cell death when they had been exposed to ultrasound.

#### Effect of shape of MSNs

The shape of MSNs influences the biodegradation and toxicity profiles on these NPs. Huang et al. designed a series of shapes (aspect ratios, 1.5, 5) of fluorescent MSNs to investigate the effects of particle shape on biodistribution, clearance and biocompatibility *in vivo*. The intravenously administrated fluorescent MSNs were mainly accumulating in the liver, spleen and lung (>80%) and there is obvious particle shape effects on *in vivo* behaviours 73. Short- and long-rod MSNs were easily trapped in the liver and spleen, respectively. Both aspect ratios MSNs with PEG surface modification had a higher content in the lung. Moreover, MSNs were mainly excreted through urine and feces, and the clearance rate of MSNs was mainly dependent on the particle shape. In addition, the short-rod NPs showed faster clearance rate than long-rod ones in through both urine and feces. Furthermore, MSNs did not result in significant *in vivo* toxicity; however, there was an induction of biliary excretion along with glomerular filtration dysfunction 73.

### *In vivo* safety of MSNs

The justification for MSNs bio-safety is extremely complicated because it varies depending on a variety of factors such as administration routes, particle weight and size, and formulations 49. Feng et al. utilized nuclear magnetic resonance (NMR)-based metabolomic analysis to investigate the biosafety of silica material. Silica NPs resulted in an increase in lipids, which could lead to membrane modification. The study's findings revealed that the toxicological effects of high doses of silica NPs can be linked to elevated levels of ATP and adenosine diphosphate (ADP), as well as glucose and amino acid utilization. Furthermore, the production of metabolic end products using silica NPs has been linked to toxicity 118.

Liu et al. studied the single- and multi-dose toxicity of hollow MSNs, administered intravenously in mice 119. For single dose toxicity, lethal dose 50 of hollow MSNs (110 nm) was greater than 1000 mg/kg, while multi-dose toxicity studies showed no death was observed when mice were exposed to hollow MSNs at 20, 40 and 80 mg/kg by continuous intravenous administration for 14 days.

Fu et al. studied the fate of MSNs (110 nm) following four different routes of administration including intravenous, hypodermic, intramuscular injection and oral 120. The intramuscular and hypodermic injections of MSNs had a low absorption rate because the NPs could cross different biological barriers into the liver. In addition, oral administration of MSNs showed high absorption into the intestinal tract and accumulated mainly in the liver while NPs, administered through intravenous injection persisted in the liver and spleen. In addition, the intramuscular and hypodermic injections of MSNs caused inflammatory response around the injection sites. It is worth mentioning that MSNs were mainly excreted through urine and feces after different exposure routes 120.

### Biodistribution of MSNs

The MSNs is excreted as either intact or degraded form mainly through hepatic and renal routes. However, there is no consensus in the literature on the exact excretion mechanism of MSNs, and more data is needed before a definitive answer can be given 121. Li et al. studied the effect of MSNs shape with different aspect ratios of 1, 1.75, and 5 on biodistribution, excretion, and toxicity after oral administration in ICR mice 121. With the increase of aspect ratio, MSNs showed decreased *in vivo* biodegradation, systematic absorption, and excretion (liver distribution and urinal excretion). Moreover, MSNs induced a shape-dependent renal damage including haemorrhage, vascular congestion, and renal tubular necrosis during urinary excretion. Furthermore, the rate of degradation of MSNs in simulated body and intestinal fluid is consistent with the biodistribution tendency 121.

Wu et al. examined the biodistribution of fluorescencent MSNs (50-100 nm) using fluorescence imaging and magnetic resonance imaging (MRI) techniques 122. Injecting MSNs into an ICR mouse resulted in 35%, 9%, 8.3%, 8%, and about 4% accumulation in liver, kidney, lung, spleen, and heart, respectively. To the best of our knowledge, MSNs have been tested *in vivo* for several DDS; however, the biodistribution of drug-loaded MSNs is still unknown.

## Patent filed for MSNs for Biomedical application

## Current perspectives

MSNs are potential candidates as DDS due to their fully tunable surface properties, pore size and volume, high loading capacity, and large surface area. MSNs entail the identification of precise targets (cells and receptors) linked to specific clinical conditions, as well as the selection of appropriate nanocarriers to achieve the desired responses at the target site while minimizing the target molecule's side effects 45,48. One can also control total loading capacity and drug release by fine-tuning surface area, pore size, and pore volume. Drug delivery for cancer treatment, bioimaging, biosensors, catalysis, and photodynamic therapy are just a few of the biological applications for MSNs.

## Future perspectives

Scalability is a key factor in industrial technology transfer, so synthesizing MSNs at a large scale could be a roadblock to commercialization 126. To ensure product reproducibility, a better understanding and control of the manufacturing process is required. Furthermore, because all drugs cannot be loaded at the same concentration, the number of MSNs may vary case wise, potentially influencing the maximum tolerated dose of MSNs. In addition, it is necessary to address the lack of in-depth understanding of the interaction between MSNs and the biological system. It is worth mentioning that the mechanism of MSNs degradation *in vivo* is still unknown.

The nanocarrier's size is also a major factor in its inability to penetrate the cell membrane. The surface functionalization of NPs is entirely responsible for oral absorption of macromolecules, proteins, and peptides in the gastrointestinal tract and delivery to the desired location 126. As a result, future advancements in MSNs in nanomedicine are still hindered by their pore size, which plays a key role in biological applications. In conclusion, future additional scientific work is still required to overcome the major challenges discussed within the review and successfully provide a new drug delivery platform in clinical practice, to eventually launch MSNs based products in the pharmaceutical market.

## Conclusion

In this review, we have provided a comprehensive account of mesoporous silica nanoparticles and their application in transdermal drug delivery that is well explained with the anatomy of the skin and different barriers in transdermal delivery. The unique properties of MSNs make them a potential candidate to be used as nanocarriers in the delivery of many therapeutic agents in a controlled manner. Different synthesis approaches such as sol-gel, evaporation-induced self-assembly together with the important parameters like silica precursor, additives, and effect of temperature that need to be considered during the production of MSNs are well explained. The review drew the attention to the role, advantages, and disadvantages of MSNs in transdermal delivery. MSNs applications, biocompatibility, and biodistribution all are discussed with relevant literature data along with the different patents filed in the field of mesoporous silica nanoparticles for biomedical applications together with current and future perspectives.

## Figures and Tables

**Figure 3:**
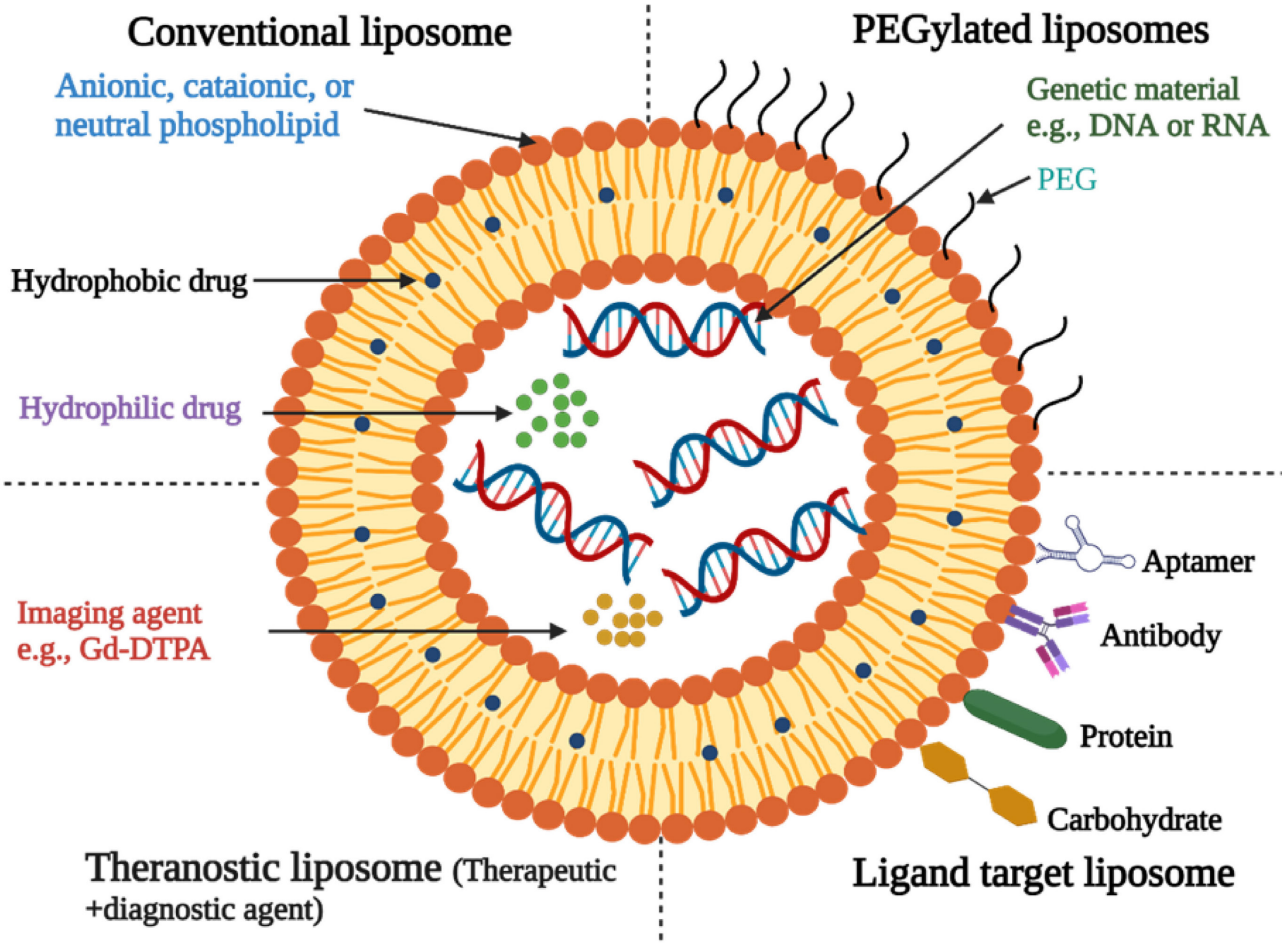
Structure of Liposomes 25. (Created with Biorender.com).

**Figure 4:**
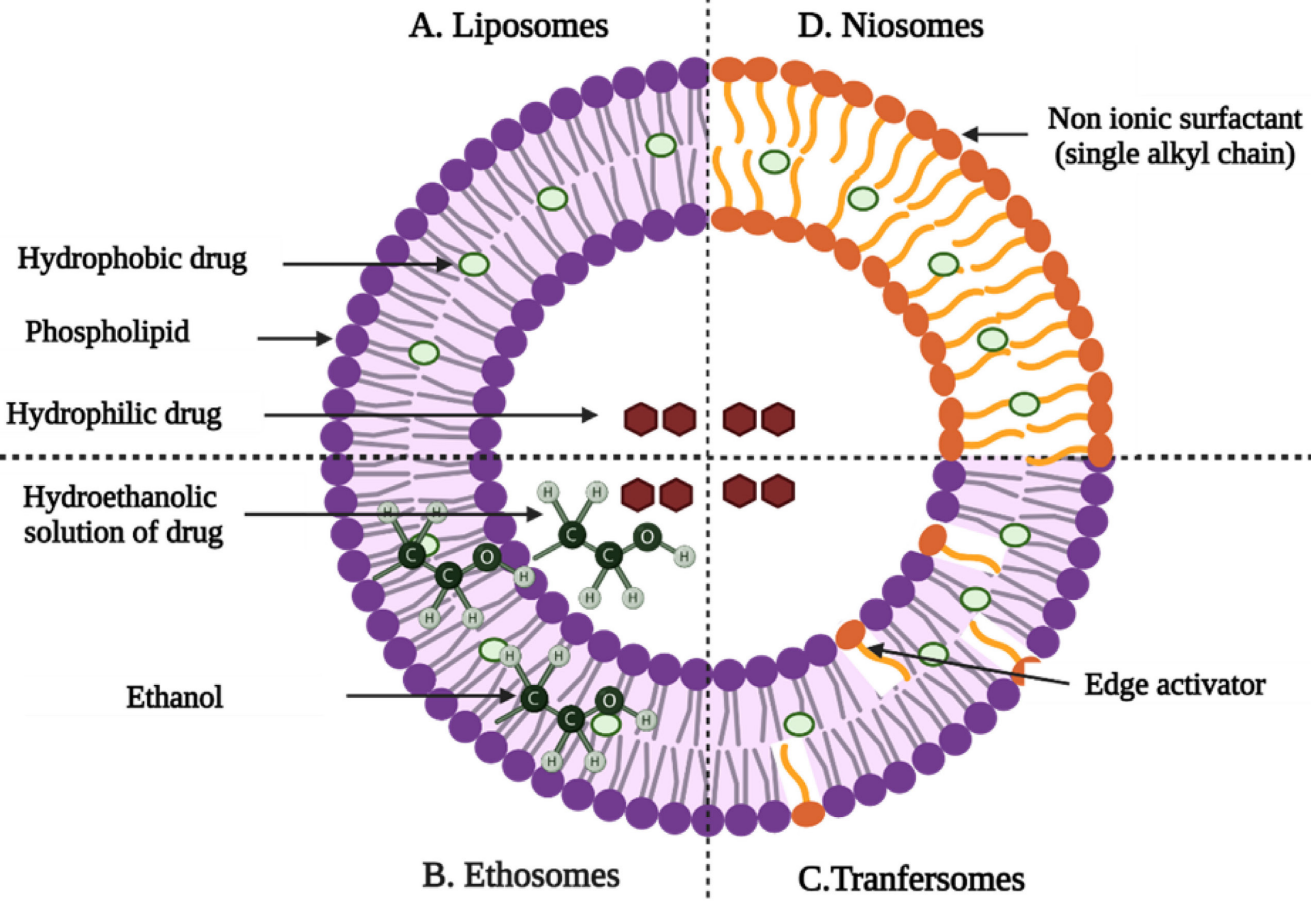
Graphical representation of vesicular drug delivery systems; (**A**) liposomes, (**B**) niosomes, (**C**) transfersomes, and (**D**) ethosomes 33 (created with Biorender.com).

**Figure 5:**
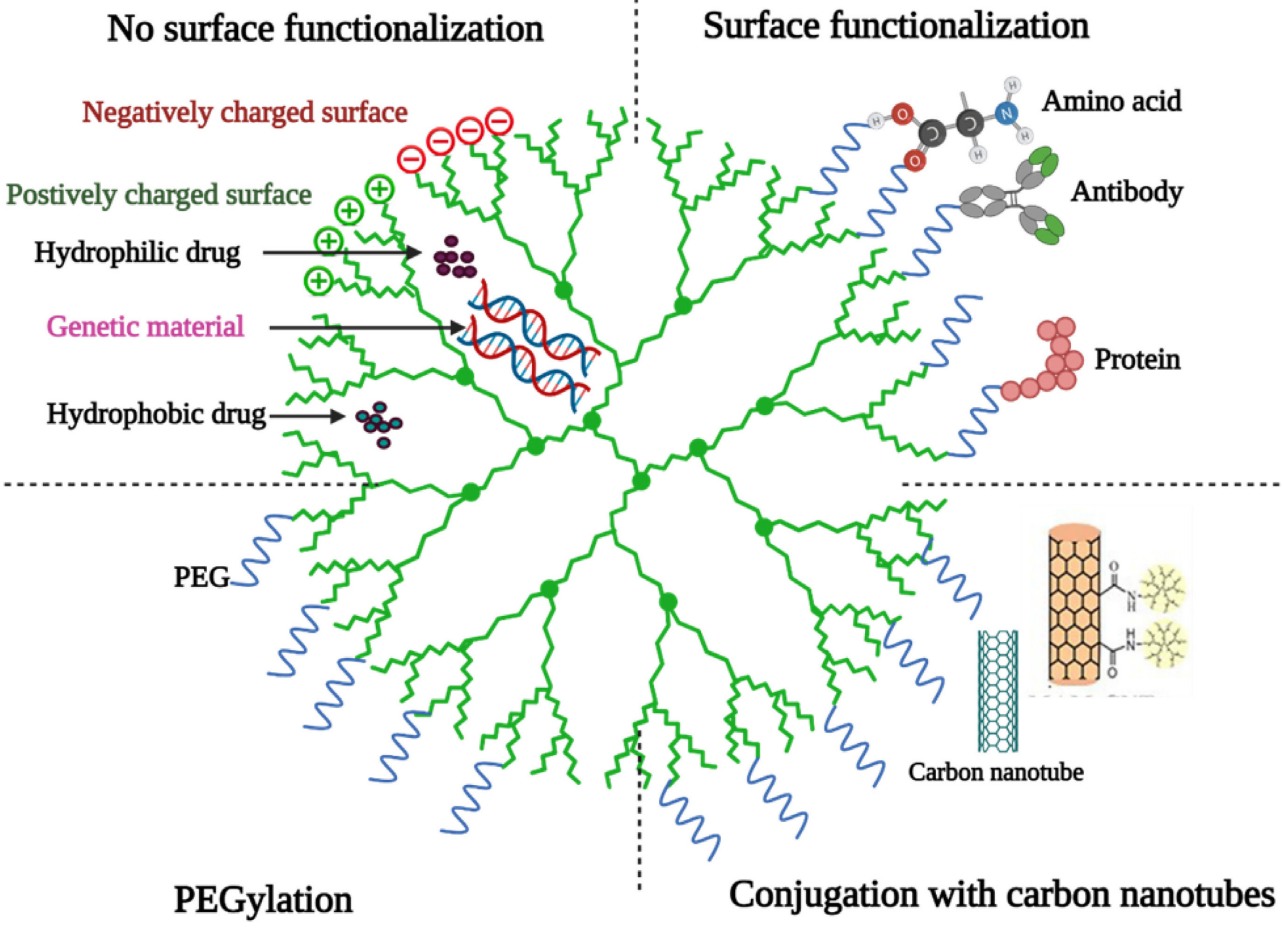
Basic structure of a dendrimer 36 (created with Biorender.com).

**Figure 9:**
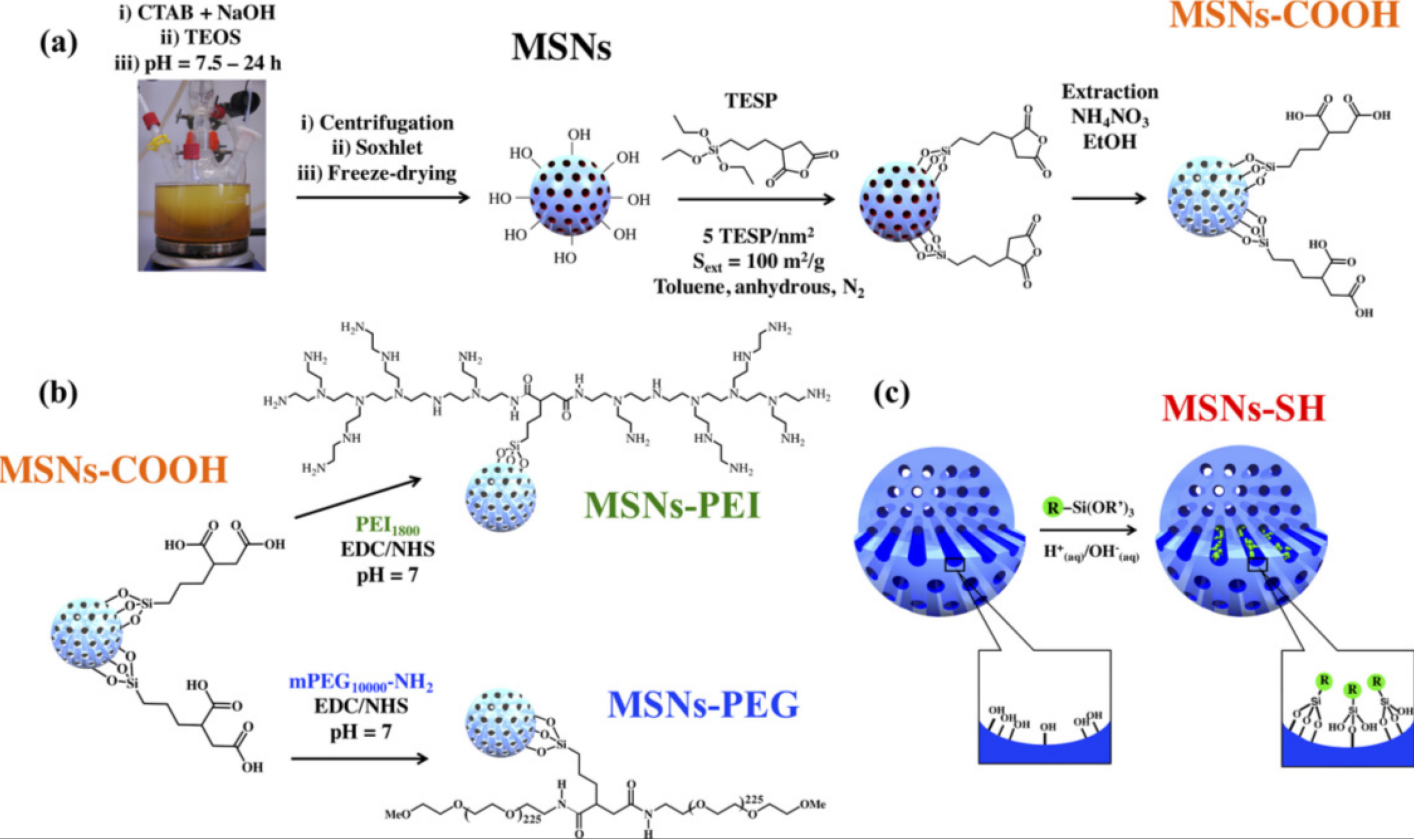
Modification of MSNs by **(a)** surface grafting of carboxylic groups (MSN-COOH) by post synthesis modification with (3-triethoxysilyl)-propylsuccinic anhydride (TESP), **(b)** coating of poly(ethylene glycol) (PEG) and polyethyleneimine (PEI) by amidation (namely, MSN-PEG and MSN-PEI, respectively), and **(c)** co-condensation with MPTES leading to MSN-SH. R =-(C_3_H_6_)SH and R′ = -(OCH_2_CH_3_)_3_. Reproduced with permission from ref 82. Copyright 2019, American Chemical Society.

**Table 1:** Some examples for transdermal delivery of drugs by various nanoparticulate drug delivery systems

Nanoparticulate formulations	Active	Nanoparticulate formulations	Active
Nanoemulsion	Glycyrrhetic acid	Transfersomes	Diclofenac Sodium
	Ketoprofen		Insulin
	Aceclofenac		Gap Junction Protein
	Nimesulide		Bleomycin
SLNs	Quercetin	Ethosomes	Methotrexate
	Betamethasone-17-valerate		Clotrimazole
	Tretinoin		Acyclovir
	Aceclofenac		Lopinavir
NLCs	Olanzapine and simvastatin	Niosomes	Acetazolamide
	Methotrexate		Ellagic Acid
	Ropivacaine		Nimesulide
	Calcipotriol and methotrexate		Capsaicin
Liposomes	Melatonin	Dendrimers	Tamsulosin
	Indinavir		Indomethacin
	Methotrexate		Diflunisal
	Estradiol		5-Fluorouracil
	Clindamycin Hydrochloride		

**Table 2:** Different types of MSNs along with their internal structure and pore size

Type	Internal structure	Pore Diameter	References
MCM-41	2D hexagonal	1.5-8	48-50
MCM-48	3D- Cubic	2-5	48-50
MCM-50	Lamellar p2	2-5	51,52
SBA 11	3D cubic	2.1-3.6	45,52
SBA 12	3D Hexagonal	3.1	45,49
SBA 15	2D Hexagonal	6-10	53
SBA 16	Cubic	5-15	45,48
KIT 5	Cubic	9.3	48
COK 12	Hexagonal	5.8	48

KIT-Korea Advanced Institute of Science and Technology, COK- Centre for Research Chemistry and Catalysis.

**Table 3:** The raw materials used during the synthesis of different MSNs 47,48

Chemical components	Function
Cetyltrimethylammonium bromide (CTAB)	Structure directing agent
Cetyltrimethylammonium chloride (CTAC)	
Non-ionic triblock copolymer	
Pluronic F123, F127	Surfactant template
Triton X-100	Surfactant
Tween 20, 40, 60, 80	
Tetraethyl orthosilicate (TEOS), Tetramethoxy silane (TMOS), Tetrakis(2-hydroxyethyl) orthosilicate (THEOS), Trimethoxyvinylsilane (TMVS), Sodium silicate	Inorganic silica source
PEO	Detergent and phase separation
Methanol	Solvent for TMOS, surfactant removal
Ethanol	Solvent for TEOS
Sodium Hydroxide, Hydrogen Fluoride, Hydrogen chloride	Catalyst
Ammonium nitrate	Surfactant removal
Hexane, water	Solvent
Polyethylene glycol	Improve biocompatibility

**Table 4:** Different patents filled for biomedical applications for MSNs

Cited Patent	Topic	Ref
US20160287717A1	Core and Surface Modification of Mesoporous Silica Nanoparticles to Achieve Cell-Specific Targeting *In vivo*.	123
US20120207795A1	Cationic polymer coated mesoporous silica nanoparticles and uses thereof	124
US8992984B1	Protocells and their use for targeted delivery of multicomponent cargos to cancer cells	125
US20160338954A1	Torroidal Mesoporous Silica Nanoparticles (TMSNPS) and Related Protocells	126
US20060018966A1	Antimicrobial mesoporous silica nanoparticles	127
